# Neuropeptide Y: An Update on the Mechanism Underlying Chronic Intermittent Hypoxia-Induced Endothelial Dysfunction

**DOI:** 10.3389/fphys.2021.712281

**Published:** 2021-08-27

**Authors:** Mei-mei Li, Yan-li Zheng, Wan-da Wang, Shu Lin, Hui-li Lin

**Affiliations:** ^1^Department of Cardiology, The Second Affiliated Hospital of Fujian Medical University, Quanzhou, China; ^2^Centre of Neurological and Metabolic Research, The Second Affiliated Hospital of Fujian Medical University, Quanzhou, China; ^3^Diabetes and Metabolism Division, Garvan Institute of Medical Research, Sydney, NSW, Australia

**Keywords:** neuropeptide Y, chronic intermittent hypoxia, vascular barrier dysfunction, vascular endothelial dysfunction, obstructive sleep apnea syndrome

## Abstract

Endothelial dysfunction (ED) is a core pathophysiological process. The abnormal response of vascular endothelial (VE) cells to risk factors can lead to systemic consequences. ED caused by intermittent hypoxia (IH) has also been recognized. Neuropeptide Y (NPY) is an important peripheral neurotransmitter that binds to different receptors on endothelial cells, thereby causing ED. Additionally, hypoxia can induce the release of peripheral NPY; however, the involvement of NPY and its receptor in IH-induced ED has not been determined. This review explains the definition of chronic IH and VE function, including the relationship between ED and chronic IH-related vascular diseases. The results showed that that the effect of IH on VE injury is mediated by the VE-barrier structure and endothelial cell dysfunction. These findings offer new ideas for the prevention and treatment of obstructive sleep apnea syndrome and its complications.

## Introduction

Hypoxia is a common clinicopathological process that can affect all organs of the body. Furthermore, it is an important cause of death from serious diseases, such as ischemic heart disease and stroke. Both human and animal studies confirm that chronic hypoxia can cause systemic inflammatory cascades, sympathetic nerve excitation, and affect the VE cell barrier. Additionally, chronic hypoxia can regulate processes, such as vascular tension; endocrine, antithrombotic, and inflammatory functions (Goligorsky, 2005; Félétou and Vanhoutte, 2006; Reitsma et al., 2007); and produce VE dysfunction (ED; Feng et al., 2012; Hung et al., 2013; McNicholas, 2019). Chronic intermittent hypoxia (IH) is more likely to cause ED than chronic persistent hypoxia (Zhu et al., 2020). Obstructive sleep apnea syndrome (OSAS) is a common disease characterized by chronic IH, and increasing evidence shows that OSAS can cause the occurrence and development of atherosclerosis (Sun et al., 2020). Furthermore, ED is a key early event in chronic IH-induced atherosclerosis (Feng et al., 2009). Chronic IH can also affect the expression of central neuropeptide Y (NPY) and the release of peripheral NPY (Sharma et al., 2009; Raghuraman et al., 2011). NPY binds to different receptors on peripheral cells to promote endothelial cell proliferation, thrombosis, vasoconstriction, and regulate cell energy metabolism (Kulkarni et al., 2000; Lee et al., 2003; Wier et al., 2009; Zhou et al., 2013). Studies clarifying the effect of NPY on the pathophysiological mechanism underlying chronic IH-induced vascular ED identify NPY as a potential target for the treatment of OSAS-related cerebrovascular diseases. This review highlights the relationship between ED and multisystem diseases, including the relationship between ED and chronic IH-related vascular diseases.

## Chronic IH and Vascular ED

### Chronic IH

Common clinical hypoxia describes the inability of the body to provide sufficient oxygenation to meet the needs of tissues when exposed to a hypoxic environment. Hypoxia can be classified as acute or chronic depending on the duration of exposure and as continuous or intermittent depending on the exposure pattern. The physiological and pathological reactions of hypoxia vary according to type (Nanduri and Nanduri, 2007). In humans, chronic IH often occurs during sleep apnea. Repeated hypoxia-reoxygenation in OSAS leads to ED through oxidative stress, inflammation, and sympathetic nerve activation among many other pathological reactions involved in disease occurrence (Prabhakar et al., 2020). Recently, a new condition associated with high altitude (i.e., long-term chronic IH) was reported in South America (Pena et al., 2020). Long-term chronic IH afflicts people that commute to work at high altitudes but live and rest at sea level. Hypoxic constriction of pulmonary vessels is the first response of pulmonary circulation to alveolar hypoxia (Hoeper et al., 2016). Among the responses, endothelial production of nitric oxide (NO), prostacyclin (PGI), and endothelin-1 (ET-1) among other vasoactive mediators are also involved in the process of pulmonary hypoxic vascular contraction (Grimmer and Kuebler, 2017).

### Vascular ED

Endothelial cells originate from the mesoderm, are arranged in a single-cell layer in the vascular cavity, and are connected *via* adhesive, tight, and gap junctions (Rodrigues and Granger, 2015). The endothelium is also covered by proteoglycans, glycosaminoglycans, glycoproteins, and glycolipids, which are referred to as the endothelial glycocalyx (GCX; Reiterer and Branco, 2020). Vascular integrity is achieved by the actions of the GCX, endothelial cells, and intercellular junctions. The endothelium plays a key role in regulating vascular permeability, vascular tension, anticoagulation, inflammation, angiogenesis, immune-response initiation, and endocrine processes. Intercellular connections regulate passive diffusion and allow the endodermis to form selective osmotic layers to maintain homeostasis (Sukriti et al., 2014). Local regulation of vascular tension by endothelial cells depends on the release of a variety of vasoactive substances, including NO, prostaglandin, ET-1, and other endothelium-derived hyperpolarization factors (Lai and Kan, 2015). Endothelial cells release tissue plasminogen activator (tPA) to prevent the development of atherosclerosis, platelet activation, and thrombus formation (Hekman and Loskutoff, 1987). The endothelium promotes angiogenesis by secreting VE growth factor (VEGF) and fibroblast growth factor (Graupera and Claret, 2018). Endothelial cells allow the initiation of adaptive immunity and local recruitment of antigen-specific lymphocytes by sensing pathogen components in the blood (Taflin et al., 2011). The abnormal function of the vascular endothelium in response to a series of risk factors (hypoxia, smoking, obesity, infection, etc.) causes pathological events, such as the progression of cardiovascular and cerebrovascular diseases, which can lead to systemic consequences.

### Relationship Between ED and Chronic IH-Related Vascular Disease

OSA is an independent risk factor for cardiovascular and cerebrovascular diseases. Furthermore, chronic IH is considered the main mediator of OSAS, and IH duration and intensity depend on the severity of OSAS. Recently, Namtvedt et al. (2013) reported that the severity of OSAS positively correlated with impaired endothelial function, and that it had an adverse effect on vascular health. Additionally, previous studies confirmed that correcting hypoxia using continuous positive airway pressure (CPAP) treatment can improve endothelial function (Wilcox et al., 2011; Xu et al., 2015). There is currently no evidence that mild OSA has any beneficial effect on endothelial function; however, there are reports that short-term, low-intensity IH or accompanying old age can to a certain extent protect against ischemic events, such as myocardial infarction and stroke (Beaudin et al., 2017). Therefore, as the initial factor of OSAS-induced atherosclerosis, ED induces vascular diseases caused by IH, which has great significance for OSAS treatment.

Vascular diseases caused by OSAS include systemic hypertension, transient ischemic attack, stroke, pulmonary hypertension, coronary heart disease, and pulmonary embolism (Bauters et al., 2016). The mechanisms underlying the increased risk of vascular disease in OSAS are thought to include sympathetic nerve activation, ED, and changes in peripheral and cerebrovascular regulation (Somers et al., 2008). Sympathetic nerve excitement, inflammation, and oxidative stress triggered by chronic IH can lead to ED and atherosclerosis.

## Mechanisms Underlying VE Injury Caused by Chronic IH

### Vascular Barrier Dysfunction

**Figure 1:**
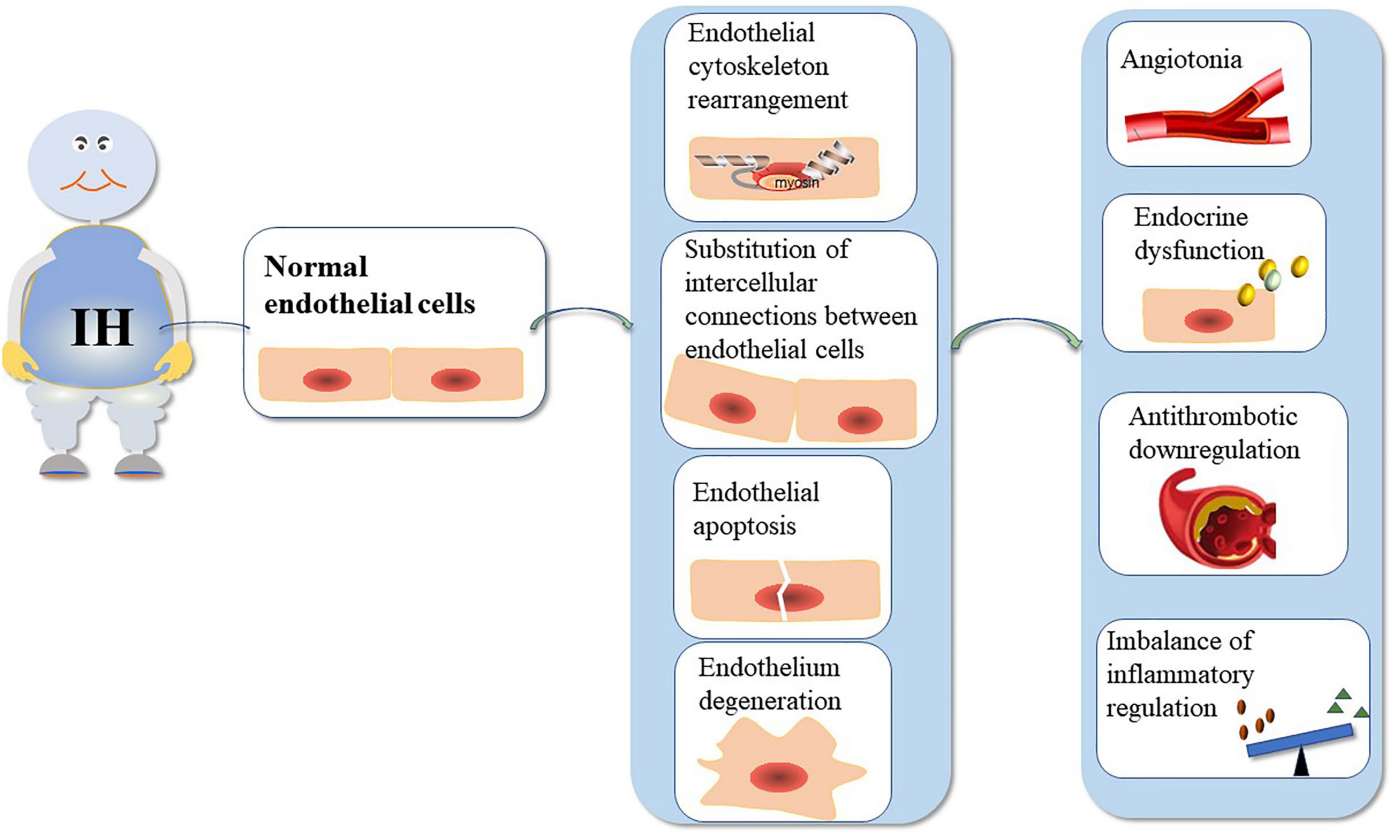
The mechanism underlying VE injury caused by chronic IH. The duration and intensity of IH are positively correlated with ED. Chronic IH-induced sympathetic nerve excitation, inflammation, and oxidative stress can lead to ED. IH can cause endothelial barrier dysfunction and endothelial cell dysfunction. ED is dependent on the integrity of the endothelial barrier.

The function of the VE barrier mainly depends on the integrity of the cytoskeleton and connexin (Dudek and Garcia, 2001). Actin and cortactin are the main endothelial cytoskeletal proteins. Additionally, IH can cause changes in the structure and function of blood vessels (Figure 1). Table 1 summarizes the mechanisms underlying VE damage caused by chronic IH.

**Table 1:** Mechanism of VE injury caused by chronic IH.

Vascular endothelial injury	Pathophysiology	Mechanism	References
Vascular barrier dysfunction	Endothelial cytoskeleton rearrangement	① IH→ROS↑→ERK1/2 and JNK phosphorylation→reorganization of cortactin and actin, stress fiber formation	Prabhakar et al., 2007; Makarenko et al., 2014
		② IH→nmMLCK↑→myosin light chain phosphorylation→cell retraction	Verin et al., 1998; Ralay Ranaivo et al., 2007; Baguet et al., 2012; Arnaud et al., 2018
	Substitution of intercellular connections between endothelial cells	① IH→influx of Ca^2+^ into endothelial cells→VEGF↑→replacement of ZO-1 and VE-cadherins	Usatyuk et al., 2006, 2012
		② IH→ROS↑→ERK1/2 and JNK phosphorylation→VE-cadherin redistributes	Dalal et al., 2020
	Endothelial apoptosis	IH→oxidative stress→ICAM-1 and VCAM-1↑→leukocyte adhesion→endothelial apoptosis↑	Zhao et al., 2018
	Endothelium degeneration	IH→TGF-β1↑→EndMT	Zhang et al., 2017; Kovacic et al., 2019
Vascular endothelial dysfunction	Dysregulation of vascular tone	IH→carotid body sympathetic tone↑/the expression ratio of arginase-1 to eNOS is out of balance→increased shrinkage capacity and vascular remodeling	Krause et al., 2015
	Endocrine dysfunction	① IH→NO, PGI, H_2_S↓/ET, TXA2↑→vasoconstriction↑	Browatzki et al., 2000
		② IH→ET-1↑→NF-κB IL-6↑→CRP↑→inflammation↑	Ivey et al., 2008
		③ IH→E-selectin, P-selectin, VCAM-1↑→AS↑	Uyar and Davutoglu, 2016;
	The antithrombotic effect was downregulated	① IH→NO↓vasodilation↓→platelet aggregation↑/leukocyte-endothelial adhesion↑	Uyar and Davutoglu, 2016; García-Ortega et al., 2019; Shahidi et al., 2020
	Imbalance of inflammatory regulation	② IH→degradation of HMW hyaluronic acid↑→TLR4/NF-κB↑	Campo et al., 2011; Zhang et al., 2019

IH, intermittent hypoxia; ROS, reactive oxygen species; JNK, c-Jun N-terminal kinase; nmMLCK, non-muscle myosin light chain kinase; VEGF, vascular endothelial growth factor; ZO-1, Zonula occludens-1; VE-cadherin, vascular endothelial cadherin; NO, nitric oxide; PGI, prostacyclin; H2S, hydrogen sulfide; ICAM-1, intercellular cell adhesion molecule 1; VCAM-1, vascular cell adhesion molecule 1; TGF-β1, transforming growth factor-β1; EndMT, endothelial cell to mesenchymal transition; eNOS, endothelial nitric oxide synthase; CRP, C-reactive protein; HMW, high-molecular-weight; TLR4, Toll like receptor 4; ET, endothelin; TXA2, thrombatin A2; NF-κB, nuclear factor kappa B; and IL-6, interleukin 6.

#### Endothelial Cytoskeleton Rearrangement

Arnaud et al. (2018) found that in the monolayer of cultured endothelial cells, 8-h of IH significantly reduced transendothelial resistance by 28%, indicating that IH leads to endothelial barrier dysfunction. Makarenko et al. (2014) found that reactive oxygen species (ROS) play a central role in promoting IH-related endothelial barrier dysfunction in pulmonary microvascular endothelial cell culture. Additionally, they reported that interval hypoxia disrupts endothelial barrier dysfunction through ROS-dependent extracellular signal-regulated kinase (ERK)1/2 activation and c-Jun N-terminal kinase (JNK)-mediated cytoskeleton and junction protein recombination (Makarenko et al., 2014). A previous study showed that ROS exposure in endothelial cells induces the redistribution of actin stress fibers and VE-cadherin to the surrounding cells, resulting in abnormal endothelial barrier function (Prabhakar et al., 2007). Moreover, non-muscle myosin light chain kinase (nmMLCK) is the only MLCK subtype expressed in endothelial cells (Verin et al., 1998), and nmMLCK phosphorylates the myosin light chain, leading to changes in cytoskeletal structure and resulting in cell retraction (Baguet et al., 2012). In lipopolysaccharide-induced sepsis models, nmMLCK induces oxidative stress and plays a key role in the destruction of the vascular barrier (Ralay Ranaivo et al., 2007). Furthermore, animal experiments by Arnaud et al. (2018) revealed that nmMLCK deletion prevented all IH-induced functional and structural changes, including correction of the integrity of the endothelial barrier. However, whether nmMLCK relies on ROS to play a role in IH-induced endothelial barrier dysfunction remains to be determined.

#### Substitution of Intercellular Connections Between Endothelial Cells

The intercellular junctions of endothelial cells are an important barrier in blood vessels and regulate the transport of water, ions, and molecules through paracellular pathways in order to maintain blood-vessel homeostasis. Zonula occludens-1 (ZO-1) and VE-cadherin represent tight junctions and adhesion junctions, respectively, that regulate endothelial cell permeability by regulating membrane adhesion of adjacent cells (Usatyuk et al., 2006, 2012). Obesity is an important risk factor for OSAS (Brant et al., 2017), and obesity hypoventilation syndrome (OHS) affects 10–20% of obese patients with OSAS. Multiple studies suggest that OHS initially interferes with endothelial homeostasis (Wang et al., 2015; Selthofer-Relatić et al., 2016; Virdis, 2016). *In vitro*, plasma exosomes from patients with severe OSAS/OHS induce ED, and isolation of exosomes from the same patient after PAP treatment and addition to brain endothelioma 3 endothelial cells significantly increased the circum-membrane-restrictive staining of VE-cadherin and ZO-1, indicating improved endothelial barrier integrity and confirming the improvement in membrane-barrier resistance observed on the ECIS assay (Bhattacharjee et al., 2018). Additionally, VEGF directly alters the intercellular junctions and actin cytoskeleton of rat endothelial cells following induction by hypoxia/reoxygenation and promotes further destruction of the blood–brain barrier (BBB), ultimately leading to neurological dysfunction (Restin et al., 2017). Moreover, Ca^2+^ is an indispensable second messenger in endothelial cells and plays a key role in regulating cell migration, angiogenesis, barrier function, inflammation, and other physiological processes. Inflammatory mediators (thrombin, histamine, and bradykinin) promote Ca^2+^ influx into endothelial cells, leading to the replacement of adhesion junctions and cytoskeletal rearrangement, thereby promoting endothelial cell permeability and contractibility (Dalal et al., 2020). Whether ROS changes the replacement of adhesion junctions and cytoskeletal rearrangement through Ca^2+^-mediated VEGF and nmMLCK expression remains to be elucidated.

#### Endothelial Apoptosis

Endothelial cell apoptosis rarely occurs in normal physiological vessels. A previous study reported that circulating endothelial cell apoptosis is an indicator of vascular injury *in vitro* (Feng et al., 2012), and an increasing number of studies show that chronic IH caused by oxidative stress can induce endothelial cell apoptosis, lead to loss of endothelial integrity, aggravate VE damage, promote the expression of redox-sensitive genes and adhesion molecules, and lead to the progression of hypoxia-induced cardiovascular diseases (Liu et al., 2018; Xiao et al., 2019). Zhao et al. (2018) found that IH-and cigarette smoke-induced emphysema can synergistically produce a greater inflammatory response and endothelial cell apoptosis in an animal model of OSAS and chronic obstructive pulmonary disease. They also showed that use of the antioxidant Tempol could antagonize these effects, as the density of apoptotic endothelial cells in OSAS patients was higher than that in non-OSAS patients. The level of apoptotic endothelial cells is associated with abnormal endothelial vasodilation. CPAP can improve hypoxia and reduce endothelial cell apoptosis; however, these studies have not yet evaluated the specific mechanism of endothelial cell apoptosis caused by chronic IH. It is of great significance to clarify the pathway of endothelial cell apoptosis for the establishment of future intervention targets. Zhang et al. (2019) used animal experiments to reveal that Toll-like receptor (TLR)4-dependent MyD88/nuclear factor kappa B (NF-κB; p65) activation plays a crucial role in the pathogenesis of hypoxia/reoxygenation-induced renal tubular dermal cell apoptosis. TLR4 regulates the expression of a large number of proinflammatory genes by controlling the fixed downstream signaling factor MyD88, which translocates and activates downstream NF-κB (p65). Based on this demonstrated relationship between chronic IH and TLR4 expression, we speculate that chronic IH also follows a conserved TLR4-dependent MyD88/NF-κB (p65)-activation pathway to induce VE cell apoptosis. This needs to be further verified by additional animal experiments and clinical translational studies.

#### Endothelial Degeneration

The endothelial-to-mesenchymal transition (EndMT) was first observed using electron microscopy in 1975. Increasing evidence shows that a portion of mesenchymal fibroblasts are derived from the endothelium, and that transforming growth factor-β1 might induce endothelial cells to proliferate into fibroblast-like cells (Kovacic et al., 2019). Endothelial cells undergo EndMT under continuous IH, which is associated with cardiovascular fibrosis. Zhang et al. (2017) found that IH can increase EndMT and the expression of prolyl 4-hydroxylase domain protein 3 (PHD3), and that IH accelerates cardiac dysfunction and increases collagen deposition *via* EndMT. Additionally, PHD3 overexpression improves cardiac dysfunction and excessive collagen deposition. Moreover, IH induces EndMT *in vitro*, causing human umbilical vein endothelial cells to appear fusiform with enhanced migration and collagen-secretion abilities. Furthermore, OSA-induced perivascular fibrosis is related to EndMT, and PHD3 overexpression might be prevented by inhibiting EndMT. These results suggest that PHD3 overexpression has therapeutic potential for disease treatment.

### Vascular ED

#### Dysregulation of Vascular Tone

The vascular endothelium regulates vascular tension by balancing the degree of vasodilation and contraction. Endothelial cells regulate vasodilation function by secreting vasoactive substances, such as NO, hydrogen sulfide, and PGI, and regulate vasoconstriction function by secreting substances, such as ET-1 and thrombatin A2 (TXA2; Krüger-Genge et al., 2019). Rats exposed to IH show elevated resting vascular tone (Phillips et al., 2006). Additionally, they observed resistance vessel remodeling in rats exposed to IH for a long period of time as evidenced by a reduced response to norepinephrine-induced constriction and acetylcholine-induced diastolic response (Phillips et al., 2004). NO plays a key role in controlling vasomotor tension, which depends on L-arginine levels. Krause et al. (2015) found that the ED in chronic IH-induced hypertension might result from an imbalance in the expression ratio of arginase-1 to endothelial NO synthase (eNOS), which results in increased vascular remodeling and contractile capacity. ET-1 plays a role by activating different receptors. ET receptor (ET_R_) A is mainly expressed in vascular smooth muscle cells (VSMCs) and mediates vasoconstriction, whereas ET_R_B is mainly expressed in endothelial myocytes and mediates vasodilation through the release of NO. Mentek et al. (2018) found that chronic IH exposure impaired the vasodilation of rat ophthalmic artery stimulated by ET_R_B, and that IH-induced oxidative stress alters the bioavailability of NO and endothelium-derived hyperpolarizing factor (EDHF), thereby changing ocular arterial reactivity. However, the importance of the contribution of EDHF to endothelium-dependent relaxation as a feature of healthy endothelium is being actively discussed.

#### ED

VE cells are also important metabolic secretory organs in the human body. VE cells regulate vasodilation by secreting vasoactive substances, such as NO, hydrogen sulfide, and PGI, and regulate vasoconstriction by secreting substances, such as ET and TXA2. ET is an active peptide secreted by VE cells and plays a major role in cardiovascular disease development. Although vasoconstriction was the first characterized effect of ET-1, there is growing evidence that ET-1 is also a potent proinflammatory cytokine involved in vascular inflammation and atherosclerosis. In human VSMCs, ET-1 activates the proinflammatory transcription factor NF-κB, which in turn induces the release of interleukin (IL)-6 and other cytokines (Browatzki et al., 2000). Furthermore, Verma et al. (2002) confirmed that the antagonistic effect of ET and the inhibitory effect of IL-6 can reduce the atherosclerotic effect of C-reactive protein. Moreover, ET-1 stimulates VSMC proliferation and migration and promotes extracellular matrix synthesis and matrix remodeling, thereby supporting its role in vascular remodeling and atherosclerosis (Ivey et al., 2008). To date, most studies have focused on the increased IH-induced vasoconstrictive response to ET-1 (Kanagy et al., 2001; Belaidi et al., 2009); however, the underlying mechanism by which ET-1 is involved in IH-associated vascular inflammation and remodeling remains unclear. Furthermore, endothelial cells secrete E-selectin, P-selectin, intercellular adhesion molecule 1, and vascular cell-adhesion molecule 1 (VCAM-1), which are involved in atherosclerotic lesion formation (Uyar and Davutoglu, 2016).

#### Downregulation of Antithrombosis

NO is a major endodermal-derived vasodilator with important vasculoprotective effects, such as the inhibition of platelet aggregation, adhesion-molecule expression, leukocyte-endothelial adhesion, and smooth muscle cell proliferation (Cohen, 1995). The vascular endothelium can also maintain thrombus balance by secreting tissue factor (TF), Von Willebrand factor (vWF), coagulation factor, and fibrinolytic components (Shahidi et al., 2020). Additionally, sleep apnea is associated with hypercoagulability, which might result from reduced NO levels and impaired vasodilation. Data from pathophysiological studies indicate that IH and sleep disruption are related to increased blood clots, ED, and venous stasis (García-Ortega et al., 2019). Moreover, a growing body of evidence suggests that OSAS is a risk factor for pulmonary embolism (Epstein et al., 2010; Arzt et al., 2012; Alonso-Fernández et al., 2013). Furthermore, studies suggest that CPAP can improve hypercoagulability and normalize the circadian rhythm of certain coagulation molecules.

#### Imbalance of Inflammatory Regulation

High-molecular-weight (HMW) hyaluronic acid is an important component of the endothelial wall and has anti-inflammatory and antioxidant properties (Campo et al., 2011). In the case of inflammation or hypoxia, HMW hyaluronic acid is degraded by hyaluronidases, such as HYAL-1, to produce proinflammatory low-molecular-weight fragments. In a clinical study of 68 OSA patients and 40 control volunteers, Meszaros et al. (2020) found that chronic hypoxia is associated with increased plasma hyaluronic acid-1 concentrations and accelerated degradation of HMW hyaluronic acid. These findings suggest that changes in hyaluronic acid metabolism are involved in the inflammatory cascade and promote ED in OSAS. In endothelial cells, hypoxia/reoxygenation exposure promotes TLR4 expression and activation of proinflammatory TLR4/NF-κB signaling, whereas TLR4 interference inhibits these effects (Zhang et al., 2017). Furthermore, IH accelerates the growth and vulnerability of atherosclerotic plaques, which might play a role in triggering the activation of proinflammatory TLR4/NF-κB signaling. These findings suggest IH as a possible risk factor for vulnerable plaques, thereby providing new insights for the treatment of atherosclerotic progression caused by OSA.

## Possible Mechanism of NPY-Induced ED

### NPY and Receptors

**Figure 2:**
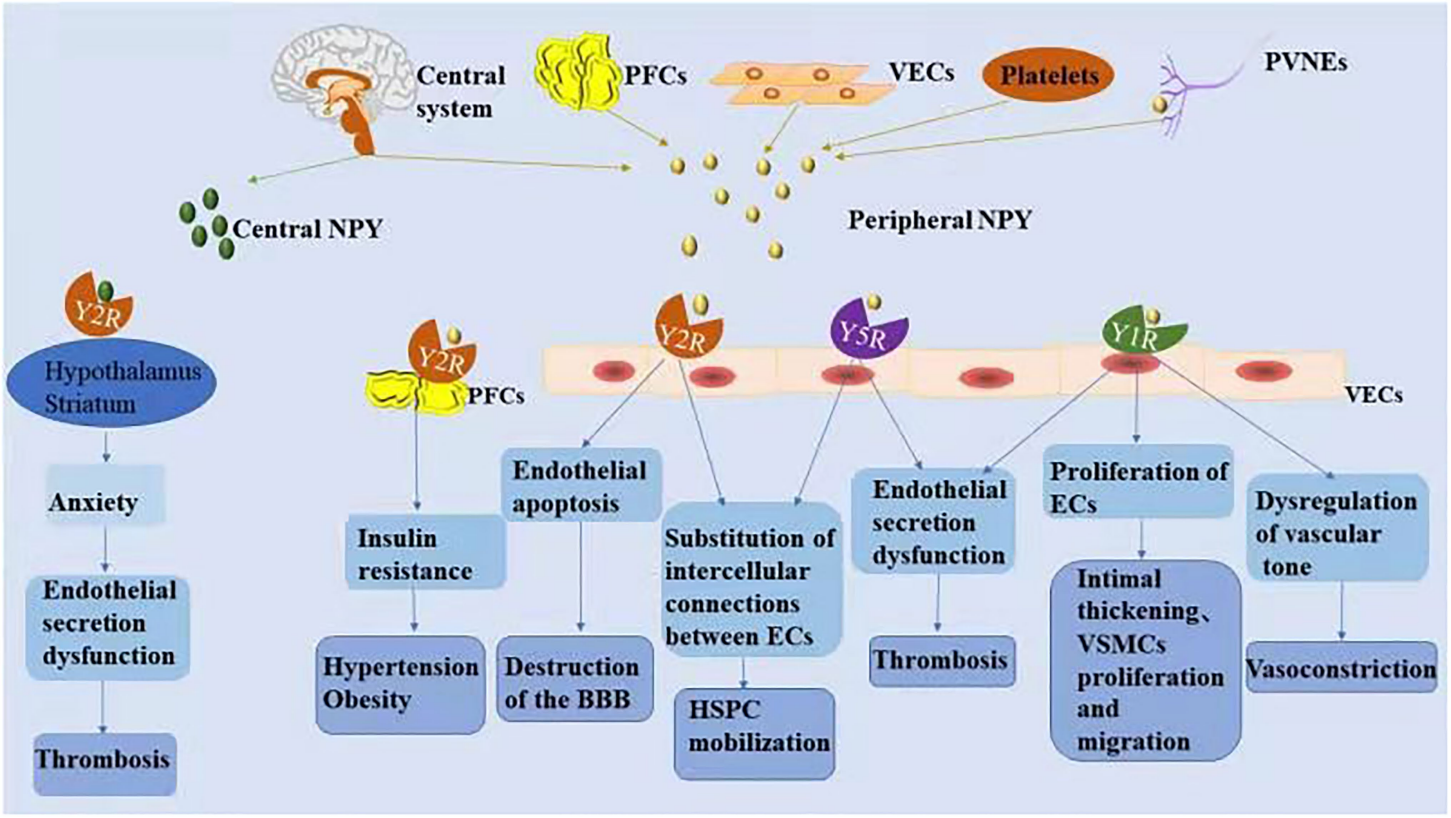
The mechanism associated with NPY-induced endothelial injury. Increased NPY level in the central and/or peripheral nervous systems is directly and indirectly involved in the process of vascular ED. NPY activates different receptors to perform various physiological functions.

NPY is a small protein-like molecule composed of 36 amino acids, widely expressed in the central and peripheral nervous systems, and participates in a variety of physiological processes by binding to receptors (Robinson and Thiele, 2017). Currently, six main subtypes of NPY receptors are found in mammals (Y1R–Y6R). Except for Y3R, these molecules are G protein-coupled receptors with different affinities and selectivity and exert different biological effects (Zhu et al., 2016). The hypothalamus is closely related to cardiovascular regulation, and its descending fibers directly reach the mediolateral column of the spinal cord to control the activity of sympathetic preganglionic neurons. NPY is secreted in the preganglionic and postganglionic sympathetic nerves and can be released into the peripheral circulation together with norepinephrine (NE) to exert a cardiovascular neuromodulatory effect (Schütz et al., 1998). Peripheral NPY is mainly secreted by peripheral vascular nerve endings, peripheral fat cells, platelets, and VE cells. Different NPY receptors on peripheral tissue cells can promote endothelial cell proliferation, thrombosis, and vasoconstriction and regulate pancreatic secretion and energy metabolism. A recent study showed that Y1R, Y2R, and Y5R are distributed on endothelial cells and mainly associated with hypertension, atherosclerosis, myocardial ischemia, heart failure, cardiac remodeling, and arrhythmia (Figure 2; Wier et al., 2009).

### NPY and ED

NPY activates different receptors to perform various physiological functions. Under various stress conditions, increases in NPY level in the central and/or peripheral nervous system is directly and indirectly involved in the process of vascular ED, thus participating in the pathophysiological process of vascular diseases (Zhu et al., 2016). The relationships between NPY and vascular ED are summarized in Table 2.

**Table 2:** The direct role of NPY in ED.

Origin of endothelial cells	Pathophysiology	Mechanism	References
Vascular endothelial cells	Substitution of intercellular connections between endothelial cells	DPP4 / CD26 cleaves NPY through CD26 signal transduction Y2R and Y5R→VE-cadherin and CD31↓→vascular permeability↑HSPC transport↑	Itkin et al., 2017; Singh et al., 2017
Brain microvascular endothelial cells	Endothelial apoptosis	METH→Y2R↑→NPY is activated by Y2R→methamphetamine-induced apoptosis and ROS formation↓	Enman et al., 2015; Ventura et al., 2020
Vascular endothelial cells	Dysregulation of vascular tone	NPY binds to the Y1R on endothelial cells to enhance the effect of NE→vasoconstriction↑→vascular stenosis and spasm↑	Li et al., 2003; Wier et al., 2009
		NPY binds to the Y1R→endothelial cell proliferation↑→intimal thickening NPY acts directly on ET-1→constricts blood vessels→ED↑	Abdel-Samad et al., 2012
EECs	Enhanced excitatory secretory coupling in EECS	NPY binds to the Y1R→cytoplasmic, nuclear calcium in EECS↑→intracellular Ca^2+^ ↑→NPY is released from cells↑	Abdel-Samad et al., 2012
EECs Vascular endothelial cells	Downregulation of antithrombosis	Y1R antagonist reduces the vWF extrusion of EECs NPY binds to the Y1R,Y5R→TXA2↑→Thrombosis NPY binds to the Y2R→tPA, clotting factor, and fibrinogen↑→Thrombosis	Mourik et al., 2013; Kleber et al., 2015; Reichmann and Holzer, 2016; Tyrrell et al., 2017
EECs	Endothelial secretion dysfunction	NPY binds to the Y5R (hLEECs)/ the Y2R,Y5R (hREECs)→ET-1↑→arrhythmia, heart failure and cardiac hypertrophy	Abdel-Samad et al., 2012

NPY, neuropeptide Y; DPP4/CD26, dipeptidyl peptidase 4/CD26; Y1R, NPY1 receptor; Y2R, NPY2 receptor; Y5R, NPY5 receptor; HSPC, hematopoietic stem cells and progenitor cells; METH, metamphetamine; ROS, reactive oxygen species; NE, norepinephrine; ET-1, endothelin-1; ED, endothelial dysfunction; EECS, endocardial endothelial cells; vWF, vascular hemophilia factors; TXA2, thrombatin A2; and tPA, tissue plasminogen activator.

#### NPY and Substitution of Intercellular Connections Between Endothelial Cells

Endothelial cells regulate the homeostasis of hematopoietic stem cells and progenitor cells (HSPCs) in the peripheral blood and are an integral part of the hematopoietic microenvironment. NPY signaling in endothelial cells modulates vascular pathways in HSPCs (Singh et al., 2017). Dipeptidyl peptidase 4/CD26 (DPP4/CD26), an enzyme that truncates NPY, induces decreased expression of VE-cadherin and CD31 at the junction of endothelial cells by altering NPY signaling, leading to increased vascular permeability and HSPC transport to peripheral blood (Itkin et al., 2017). Additionally, selective Y2R and Y5R antagonists restore vascular integrity and restrict HSPC mobilization, suggesting that the enzyme-controlled vascular pathway specifically opens through CD26 signaling involving Y2R and Y5R to cleave NPY. Mice lacking CD26 or NPY show impaired HSPC transport, which is restored with truncated NPY treatment (Singh et al., 2017); therefore, these results indicate that the CD26-mediated NPY axis might be a potential drug target for various immune stressors regulating barrier integrity on VE cells.

#### NPY and Endothelial Apoptosis

Endothelial cells are the anatomical basis of the BBB. Human microvascular endothelial cells express both Y1R and Y2R receptors (Enman et al., 2015). Ventura et al. (2020) used a human brain microvascular endothelial cell line (HCMEC/D3) to simulate an *in vitro* BBB model and found that only Y2R was upregulated during metamphetamine (METH) exposure. NPY is activated by Y2R and plays a protective role in METH-induced apoptosis and ROS formation in endothelial cells, suggesting the Y2R subtype as a promising target for the prevention of METH-induced neurovascular dysfunction.

#### NPY and Dysregulation of Vascular Tone

As the main peripheral vasoconstrictive neurotransmitter, NPY binds to Y1R on endothelial cells under pathological conditions to enhance the effect of NE, plays a role in vasoconstriction, causes local vascular stenosis and spasm, and then promotes endothelial cell retraction and interruption (Wier et al., 2009). Additionally, NPY binds to Y1R on endothelial cells, promotes endothelial cell proliferation, and plays a key role in intimal thickening (Li et al., 2003). NPY constricts blood vessels directly or through molecules, such as ET-1, causes vascular ED, promotes VSMC proliferation and migration, and converts the contractile phenotype of VSMCs into a synthetic phenotype with proliferative function (Abdel-Samad et al., 2012). The presence of the cardiac active factor NPY and its receptor Y1R in endocardial endothelial cells (EECs) was confirmed using three-dimensional confocal microscopy. By binding to its receptors, NPY can induce an increase in cytoplasmic and nuclear Ca^2+^ in EECs, after which NPY is released from these cells to regulate the excitation-secretion coupling of EECs and the excitation-contraction coupling ability of cardiomyocytes and VSMCs, thereby indirectly regulating cardiac function and remodeling (Abdel-Samad et al., 2012).

#### NPY and Downregulation of Antithrombosis

EECs are more prone to thrombosis with the progression of heart failure (Saric et al., 2016). EECs are attached to the sympathetic nerve of the heart, and neuropeptides, such as galanin and NPY, are released jointly by sympathetic nerve endings (Herring et al., 2012). Thrombin (FIIa) at the surface of the endothelial tube cavity converts fibrinogen to fibrin and promotes the release of vascular hemophilia factors (vWF) from endothelial cells (Mourik et al., 2013; Kleber et al., 2015). Clusters of vWF gather on the endothelial surface to help platelets adhere. Tyrrell et al. (2017) showed that glycerin and a Y1R antagonist reduced vWF-mediated extrusion of EECs. Additionally, nicotine exposure experiments showed that nicotine promotes NPY expression and induce ED (Hiremagalur and Sabban, 1995; Huang and Winzer-Serhan, 2007). Nicotine increases the expression of TF and TXA2 and decreases the expression of prostaglandin I-2, leading to platelet adhesion and aggregation by affecting the secretory function of endothelial cells (Amadio et al., 2015). Moreover, Csordasa and Bernhard (Csordas and Bernhard, 2013) observed that NPY promotes the release of TXA2 from endothelial cells by activating Y1R and Y5R, thereby inducing thrombus formation. Furthermore, nicotine and NPY combine to promote pathological thrombosis. NPY also induces anxiety by activating Y2R in the hypothalamus and striatum (Reichmann and Holzer, 2016), with anxiety increasing the risk of thrombosis, which is promoted by inducing endothelial cells to release tPA, clotting factor, and fibrinogen.

#### NPY and Dysfunctional Endothelial Secretion

In cultured human EECs, NPY treatment promotes the release of ET-1 from left and right (LEECs and REECs, respectively); however, the type of NPY receptor involved might be different (Abdel-Samad et al., 2012). The effect of NPY on ET-1 secretion of human (h) LEECs resulted only from Y5R activation, whereas the effect of NPY on ET-1 secretion of hREECs mainly resulted from the activation of Y2R and part of Y5R. EECs play an important role in cardiac pathological processes, such as excitation-contraction coupling, arrhythmia, cardiac hypertrophy, and heart failure, by secreting cardiac active factors, such as NPY and ET-1 (Jacques et al., 2017).

#### Indirect Effects of NPY

Increased NPY level in the central and/or peripheral nervous system is associated with dyslipidemia, hypertension, obesity, diabetes, and impaired glucose tolerance, all risk factors for atherosclerotic cardiovascular disease (Sun et al., 2017). During stress, NPY released from sympathetic nerves participates in adipose tissue insulin resistance through Y2R in visceral adipose tissue, promotes food intake and fat storage, and enhances macrophage-mediated inflammatory responses (Kuo et al., 2007). Additionally, NPY increases the dependence on nicotine, which indirectly aggravates ED (Robinson and Thiele, 2017). These pathological processes promote oxidative stress, inflammatory response, blood-flow disorders, and reduce NO bioavailability, all of which eventually induce ED.

## Involvement of NPY in the Mechanism Underlying IH-Induced ED

**Figure 3:**
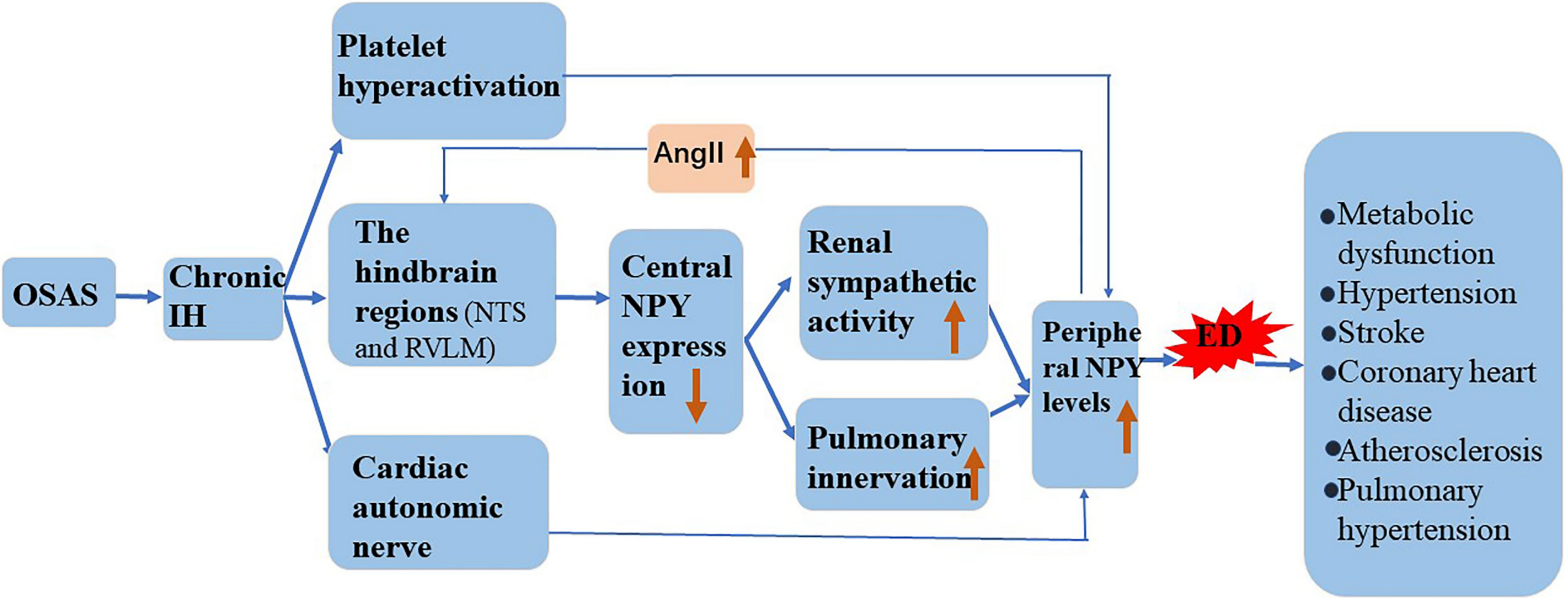
Involvement of NPY in the mechanism underlying IH-induced ED. Chronic IH affects central NPY expression and peripheral NPY levels. NPY is involved in the pathogenesis of atherosclerosis, hypertension, stroke, coronary heart disease, and metabolic dysfunction by increasing ED.

Chronic IH promotes NPY expression, and the induction of ED by NPY is similar to that of chronic IH. In the following sections, we attempt to elaborate on the role of NPY in chronic IH-induced ED and its possible mechanism (Figure 3).

### NPY and CIH

Hypoxia induces the release of NPY in mammals (Ali and Bhargava, 2017), and chronic IH affects central NPY expression and peripheral NPY levels. Shobatake et al. (2018) demonstrated that IH reduces NPY expression in the central nervous system and weakens the inhibitory effect of sympathetic excitement. NPY is mainly distributed in peripheral sympathetic nerve fibers, especially those in and around the cardiovascular system. Animal experiments confirmed that hypoxia directly stimulates the release of large amounts of NPY from the autonomic nervous system (McDermott and Bell, 2007). We speculate that in response to chronic IH, central NPY expression decreases, and peripheral sympathetic nerve activity increases. Additionally, hypoxia causes sympathetic excitation and promotes the release into the circulation of perivascular neurons from postganglionic sympathetic nerves. Chronic IH causes overactivation of the hindbrain regions that control sympathetic outflow, such as the nucleus solitudes and ventrolateral medulla oblongata, and promotes increased sympathetic outflow (Shell et al., 2016). Moreover, increased renal sympathetic activity activates the RAS system, leading to elevated circulating angiotensin II (AngII), and an increase in circulating AngII reportedly further increases central sympathetic outflow (Kim et al., 2018). The pulmonary artery is innervated, and NPY/Y1R mediates the vasoconstriction and proliferation of pulmonary hypertension. Furthermore, in a mouse model of chronic hypoxia, the expression of NPY and Y1R was upregulated in lung tissue (Crnkovic et al., 2014). Platelet hyperactivation during chronic IH and activated platelets might further increase the circulating concentration of NPY (Gabryelska et al., 2018).

### Possible Mechanism of NPY Involvement in IH-Induced ED

NPY participates in the pathogenesis of atherosclerosis by aggravating ED, VSMC growth, foam cell formation, and platelet aggregation, which are major pathogenic processes in cardiovascular disease (Zhu et al., 2016). However, whether vasoconstriction, vascular remodeling and vascular ED caused by chronic IH are mainly caused by the release of NPY has not yet been determined. Although OSAS causes VE damage, platelet activation increases in patients and promotes platelet aggregation in the damaged intima (Kontos et al., 2020). Additionally, NPY expression is significantly elevated in activated platelets and associated with a cycle of endothelial injury (Ruohonen et al., 2009). Moreover, ED reportedly causes increased NPY expression, further increasing the inflammatory/immune response associated with NPY in the endothelium (Choi et al., 2019). Whether NPY-mediated endothelial function forms a positive feedback loop after sympathetic excitation and ultimately leads to the deterioration of atherosclerosis remains to be clarified.

### Effect of NPY on the Pathogenesis of Chronic IH-Related Vascular Diseases

OSAS is associated with cardiovascular and cerebrovascular diseases, including atherosclerosis, hypertension, stroke, coronary heart disease, and metabolic dysfunction, contributing to an overall increase in cardiovascular mortality. ED is the primary cause of atherosclerosis through complex interactions among lipoprotein accumulation, inflammatory infiltration, foam cell formation, and smooth muscle cell changes (Jiang et al., 2017). Decreased NO bioavailability leads to ED. Experiments in eNOS-deficient mice confirmed that ED induces upregulation of NPY expression (Choi et al., 2019). Additionally, inflammatory protein 3 amplifies vascular inflammation by increasing macrophage chemotaxis *via* the NPY/NPY receptor axis, thereby increasing the inflammatory/immune response in the endothelium. Furthermore, NPY induces lipid uptake of VSMCs and triggers the formation of smooth muscle foam cells (Tan et al., 2018).

OSAS is the main cause of secondary hypertension, with a previous study confirming a close and independent relationship between secondary hypertension and nocturnal blood pressure instability (Parati et al., 2013). Sympathetic nerve excitation is the main pathophysiological mechanism underlying this relationship. When the apnea period is prolonged, the partial pressure of oxygen in the arteries decreases and carbon dioxide gradually increases in a process that stimulates chemoreceptors and causes sympathetic nerve excitement and release of NE. NPY is an important sympathetic neurotransmitter in peripheral blood vessels and released together with NE. The adrenal medulla is a component and effector organ of the sympathetic nervous system. NPY and NE are synthesized and stored in large, dense core vesicles of the adrenal medulla. Animal studies show that IH increases NPY synthesis in the adrenal medulla of rats, thereby inducing an increase in blood pressure (Raghuraman et al., 2011). Moreover, an impaired baroreflex, activation of RAS, dysfunctional NO metabolism in the vascular endothelium, and related downstream consequences leading to arterial vasoconstriction also play a role in OSAS-induced hypertension (Ryan et al., 2007; Zalucky et al., 2015; Crinion et al., 2017). However, the full mechanism of ED-induced OSAS-mediated hypertension remains unclear. Moreover, whether NPY is directly involved in the increase in blood pressure induced by IH-induced ED has not yet been determined. Thus, NPY and its receptors might be potential therapeutic targets for the treatment of OSAS-related hypertension in the future.

## Conclusion

The nervous system plays an important role in vascular function and participates in its regulation. Although the effect of NPY on vascular ED has recently attracted attention, its specific role in ED pathogenesis remains unclear. In particular, whether NPY and its receptor are involved in OSAS-induced ED has not yet been determined. Furthermore, the factors that increase the risk of OSAS-induced vascular disease are complicated and require further identification. The effects of IH on endothelial function are limited by the severity of OSAS, and the animal models that mimic OSAS are diverse. Animal experiments and clinical studies are indispensable to investigating the contributions of NPY in OSAS-induced ED. Genetic studies have supported the association between NPY polymorphisms and an increased risk of cardiovascular diseases (Masoudi-Kazemabad et al., 2013; de Luis et al., 2017). Taken together, the application of NPY genomics to the study of associated vascular diseases may provide new ideas for the prevention and treatment of OSAS and its complications in the future.

## Author Contributions

M-mL, Y-lZ, W-dW, H-lL, and SL were involved in the genesis of the review topic and reviewed, edited, and approved the manuscript. M-mL drafted the manuscript. All authors contributed to the article and approved the submitted version.

## Conflict of Interest

The authors declare that the research was conducted in the absence of any commercial or financial relationships that could be construed as a potential conflict of interest.

## Publisher’s Note

All claims expressed in this article are solely those of the authors and do not necessarily represent those of their affiliated organizations, or those of the publisher, the editors and the reviewers. Any product that may be evaluated in this article, or claim that may be made by its manufacturer, is not guaranteed or endorsed by the publisher.
